# Hybrid Decision-Level Fusion of CNN-Based Deep and Handcrafted Features for Colon Cancer Classification

**DOI:** 10.3390/jimaging12080368

**Published:** 2026-08-09

**Authors:** Simona Moldovanu, Adina Cocu, Diana Stefanescu, Cătălin Anghel

**Affiliations:** 1 Department of Computer Science and Information Technology, Faculty of Automation, Computers, Electrical Engineering and Electronics, “Dunarea de Jos” University of Galati, 800146 Galati, Romania; diana.stefanescu@ugal.ro (D.S.); catalin.anghel@ugal.ro (C.A.); 2 The Modelling & Simulation Laboratory, “Dunarea de Jos” University of Galati, 47 Domneasca Str., 800008 Galati, Romania

**Keywords:** hybrid decision level, late fusion, handcrafted features, explainable methods

## Abstract

In recent years, there has been increased attention on classifying histopathological images through hybrid decision-level fusion, and the challenge of exploring data fusion to improve classification accuracy in colon cancer has become significant. This study introduces new elements by incorporating various deep learning (DL) architectures, including EfficientNetB0, DenseNet121, ResNet101V2, NASNetMobile, MobileNetV2, and VGG16 Convolutional Neural Networks (CNNs), as well as Random Forest (RF) and Histogram Gradient Boosting (HGB) Machine Learning (ML) algorithms, along with the original dataset. The proposed hybrid decision-level fusion approach analyzes the LC25000 dataset’s colon histopathological images and handcraft features (HFs) to improve predictive performance. The HFs such as entropy, the Gini index, and the radius of gyration from adenocarcinoma and benign colon tissue (CC) were extracted. The prediction of the proposed models leveraging late fusion was conducted by classifying both deep and HFs. During the experiments, it was demonstrated that the combination of ResNet101V2 with RF classifier and all HFs yielded greater accuracy and consistent performance. The achieved performance metrics include accuracy of 94.1%, F1-score of 94%, Matthews Correlation Coefficient (MCC) of 88.3%, and an area under the curve (AUC) of 0.979. To explain and interpret the decisions made by the DL models, the explainable methods SHapley Additive exPlanations (SHAP) and Gradient-weighted Class Activation Mapping (Grad-CAM) were utilized.

## 1. Introduction

According to statistics from the World Health Organization, colon cancer (CC) ranks as the second leading cause of death. In 2020, over 1.9 million cases of CC were recorded, with more than 930,000 deaths resulting from the disease. By 2040, statistics indicate that CC will claim 1.6 million lives annually, representing a staggering 73% increase. Furthermore, the number of new cases is expected to reach 3.2 million, representing a 63% jump. The frequency and death rates show notable differences based on regions, with CC being most prevalent in Europe, Australia, and New Zealand.

Histopathological images serve as the benchmark for diagnosing CC. Pathologists analyze tissue samples from biopsies using a microscope, which allows them to gather essential insights that inform treatment decisions [1]. This paper studies benign colon conditions and adenocarcinoma; polyps and fatty tumors fall into the first category, while adenocarcinoma, the most common type of colorectal cancer, originates in the mucus-producing gland cells that line the colon, grows uncontrollably, invades the colon wall, and spreads fast [2].

Researchers have been conducting analyses of histopathological images (HI) using a diverse range of deep learning methods. Recently, machine learning (ML) techniques have gained traction in the classification of cancer types. Naik et al. [3] employed Random Forest (RF), Support Vector Machine (SVM), and k-Nearest Neighbors (KNN) to analyze benign and malignant breast cancer. Tătaru et al. [4] proposed the PyCaret Auto Machine Learning tool for classifying features extracted from HI related to diabetes mellitus pathology. In deep learning models, pre-trained or custom-built convolutional neural networks (CNNs) and transformers play a crucial role in the analysis and classification of HI. The integration of various data types and deep learning models has led to the emergence of a new paradigm known as multimodal learning.

Hybrid decision-level fusion refers to how multiple deep learning models are combined and learned. In medical AI, the main multimodal learning categories are early, intermediate, and late fusions [5]. Huang et al. [6] proposed a multimodal DL model based on the ResNet network directly from histopathological images and clinical data to predict tumor mutational burden. A robust model for colorectal cancer, based on a transformer-based survival analysis model (TransSurv) and deep features extracted from histopathological images, genomic data, and clinical information, was implemented by Lv et al. [7]. Kılıç and Doğan [8] developed an innovative DL framework that enhances skin cancer diagnosis from images. Their approach incorporates transfer learning, data-balancing techniques (including SMOTE and augmentation), model pruning, and an enhanced pooling method. This strategy achieves an accuracy of 91.52% while also reducing model complexity, making it a successful and efficient tool for clinical applications.

Explainable AI (XAI) techniques aim to clarify the opaque nature of complex algorithms, elucidate the rationale behind diagnostic decisions, and improve the interpretability of diagnostic outcomes [9]. The XAI methodology uses SHAP to make model decisions clearer and show which features are most important [10]. Multimodal learning in medical AI is used to explain the classification of metadata of features extracted from images. Grad-CAM functions as a foundational tool that highlights the most critical regions of an image employed in a deep learning model’s decision-making, hence improving the interpretability and confidence in results. It is utilized in diverse computer vision processes encompassing explanation and classification [11]. The Grad-CAM is useful for highlighting tumor regions [12], squamous cell carcinoma [13], or colon cancer diagnosis [14].

This study implemented a comprehensive experimental methodology for classifying benign and adenocarcinoma of the colon through late hybrid decision-level fusion. The approach utilized six CNNs, two ML classifiers, three HFs, and two XAI methods. We made the following contributions to achieve optimal results and improve medical workflows.

(1) Unlike many approaches that depend only on deep features, we introduced an advanced hybrid decision-level fusion strategy that integrates six pretrained CNN models, two ML algorithms and two CC classes. This approach combines EfficientNetB0, DenseNet121, ResNet101V2, NASNetMobile, MobileNetV2, and VGG16 CNNs with RF and HGB ML algorithms.

(2) Redundancy in the LC25000 dataset was addressed through the elimination of duplicate images using a perceptual hashing approach.

(3) The performance of CC classification was evaluated through an ablation study conducted on all pretrained CNNs used in this study. The models were first tested independently and then integrated into a hybrid decision-level fusion framework. Appendix A presents the supplementary experimental results obtained from the ablation analysis, including six combinations of the three handcrafted features. The results obtained in the ablation process with dumbbell plots and the Robustness Index (RI) were analyzed.

(4) The histopathological images (HI) exhibit a spatial distribution of patterns. For their meticulous analysis, we selected relevant features that characterize histopathological images. In this sense, we computed Shannon entropy (SE), Gini index (GI), and Radius of gyration (RG), which together form the HFs set for pattern analysis. Before feeding the CNNs, we statistically analyzed the handcrafted features with violin plots for hybrid decision-level fusion to assess their consistency.

(5) The late fusion of deep features and HFs was successfully achieved during the fine-tuning process.

(6) The classification evaluation was conducted using quality metrics such as accuracy, F1-score, Matthews correlation coefficient (MCC), and area under the curve (AUC).

(7) Extensive experimentation was carried out to establish the model predictions; their contribution was emphasized with SHAP and Grad-CAM explainability AI methods.

The remainder of this paper is structured as follows. In Section 2, the current cutting-edge studies are analyzed. Section 3 explains the methodology of the proposed work. Section 4 shows the materials and methods. Results and discussions are explored in Section 5. Finally, Section 6 concludes the study.

## 2. Related Work

This section aims to highlight the trends and gaps related to benign and adenocarcinoma CC. We have chosen cutting-edge papers from the past five years that concentrate on histopathologic images or hybrid decision-level fusion. Specifically, we conduct a thorough investigation to identify the deep learning models used, the application of explainable artificial intelligence, the datasets employed, and the value of quality metrics. Table 1 presents, besides the mentioned elements, the references and publishing year.

The selected studies combine two deep learning models applied to medical datasets [15,16,17,18,19,20,21,22,23,24,25]. The authors in [18,19,21] implemented the XAI method to explain the results. The studies [4,26] introduced custom-built CNNs, whereas [15,16,17,18,19,20,21,22,23,24,25] utilized pre-trained CNNs.

The common gap in studies in the scientific literature is that these focus on the same category of deep features, or deep and clinical features. Instead, studies often neglect handcrafted or radiomic features that express the spatial arrangement of pixels and characterize the image pattern.

Adding HFs could greatly improve understanding of the complex relationships in the data, which would lead to more accurate predictions and insights. By integrating these features into hybrid decision-level fusion frameworks, researchers may uncover new patterns that are crucial for advancing diagnostic and treatment strategies. The particular gap in cutting-edge papers is the omission of XAI methods. This omission can lead to a lack of transparency in decision-making processes, ultimately hindering trust and understanding learning models.

## 3. Methodology

**Figure 1:**
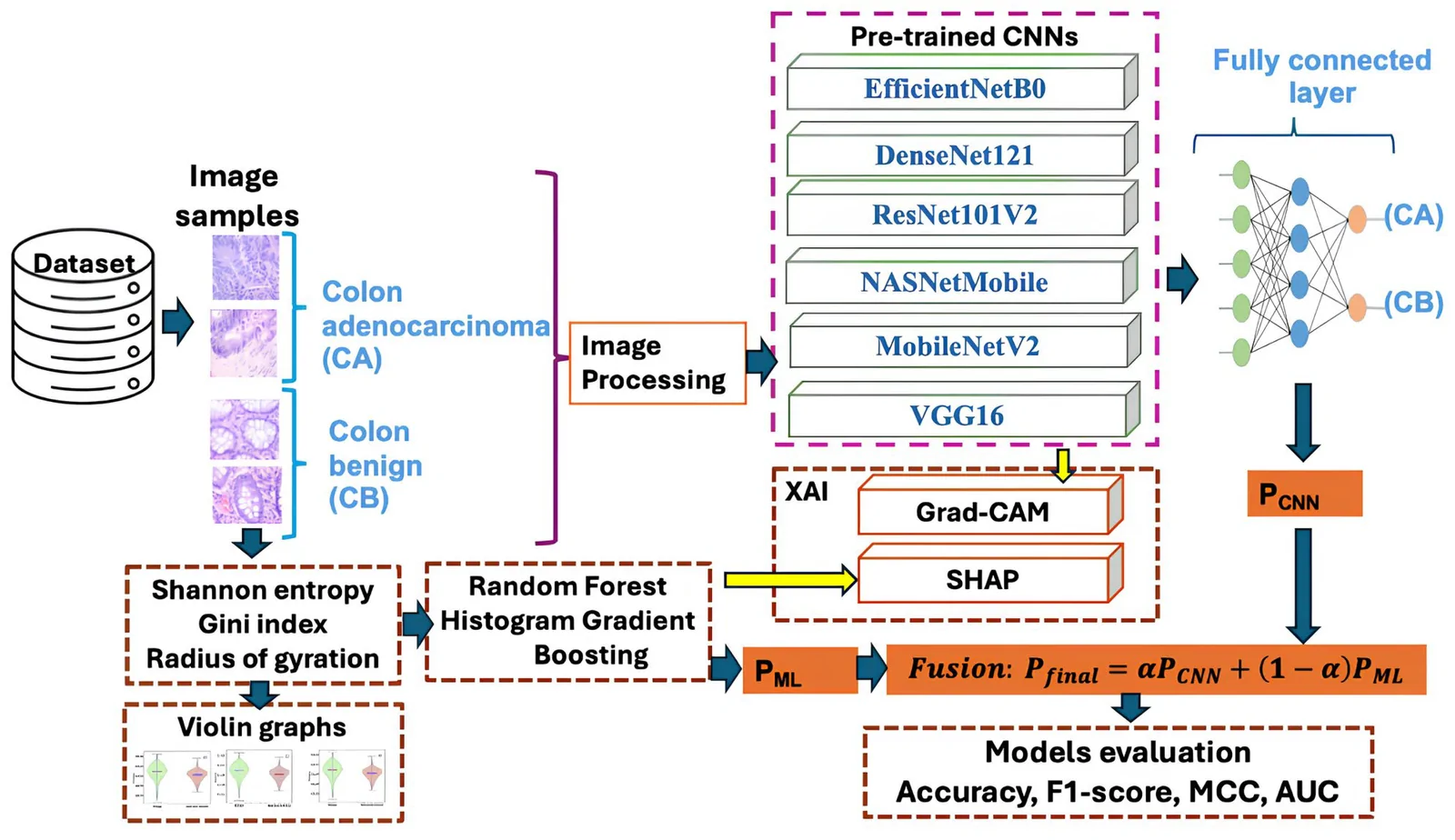
The detailed structure of the proposed study.

The whole hybrid decision-level fusion process for a salient classification of CC is summarized step by step in this section, and the blocks are shown in Figure 1. The initial step in this study was to select the LC25000 dataset. The first block depicts some samples of benign and adenocarcinomas of the colon that belong to this dataset.

The preprocessing step involved resizing the images to a size of 224 × 224 pixels to ensure compatibility with EfficientNetB0, DenseNet121, ResNet 101V2, NASNetMobile, MobileNetV2, and VGG16 pre-trained convolutional neural networks (CNNs); and augmentation with random rotation and color jitter methods.

A fully connected layer is linked to the final node of each pretrained CNN. This layer learns task-specific decision boundaries by mapping extracted features to logits, while the Softmax layer transforms those logits into interpretable class probabilities.

In parallel with deep features, the Shannon entropy, Gini index, and radius of gyration were extracted from each histopathological image, interpreted using violin graphs, and classified using RF and HGB classifiers.

The main step is late fusion; it combines the prediction obtained from deep features, stored in the vector P_CNN_, with the prediction obtained with ML algorithms and stored in P_ML_. The final fused probability used for the decision P_final_ is established in accordance with the fusion weight α∈[0,1].

The XAI block contains Grad-CAM, which allows for visualizing which parts of an image a CNN focuses on when making a prediction by highlighting the regions that significantly influenced a specific class decision. By clarifying the reasoning behind a model’s decision, SHAP enhances transparency in ML decisions on handcrafted features, thereby building trust. The final combination of six CNNs with each ML algorithm was assessed in a binary classification using accuracy, F1-score, MCC, and AUC quality metrics.

## 4. Materials and Methods

### 4.1. Image Dataset

**Figure 2:**
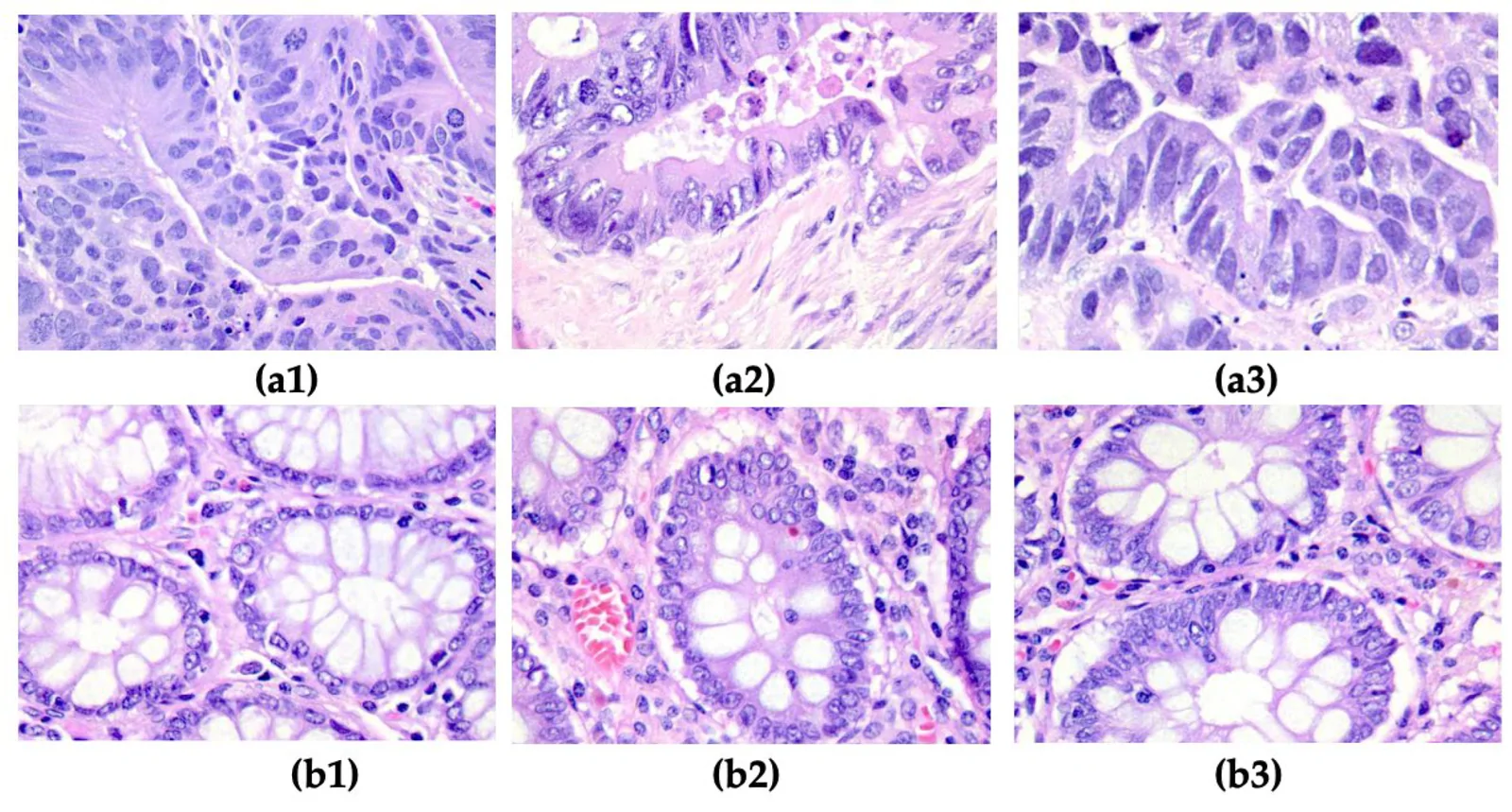
Samples from LC25000; (**a1**–**a3**) Colon adenocarcinoma; (**b1**–**b3**) Colon benign tissue.

In the proposed study, the LC25000 colon histopathological image dataset was utilized. This dataset contains histopathological images acquired from colon tissues. All images are in JPEG file format and have a resolution of 768 × 768 pixels. It includes 5000 images of benign colon tissues and 5000 images of colon adenocarcinomas. The repository of images can be accessed at https://github.com/tampapath/lung_colon_image_set; the last access date was 22 December 2025 [28]. Figure 2 presents samples of adenocarcinoma and benign colon tissue.

The second dataset was derived from LC25000, where features such as Shannon entropy, Gini index, and radius of gyration were extracted from each image. These textural features, which characterize the patterns in the images, are stored in a repository accessible at https://github.com/simonamoldovanu/pattern_features_lung, the last access date was 15 July 2026. The total number of records in this dataset is 10,000, corresponding to the total number of images. The training and testing stages use 70% and 30% of each dataset, respectively.

We used a perceptual hashing (pHash) method to remove duplicate images from the LC25000 dataset to minimize data leakage and redundancy. Based on the content of images, perceptual hashing creates a condensed image representation. Using the Hamming distance measure, a perceptual hash was computed for every image and compared to the hashes of all previously stored images. Images that had a Hamming distance below a certain point were eliminated because they were deemed to be near duplicates. The Hamming threshold was set at 4 for a strict duplicate removal. Under these circumstances, two images were eliminated from the entire LC25000 dataset. Further, the duplicate removal was done before the train/test split, ensuring that no duplicate or near-duplicate images could leak into either of these sets. After duplicate removal, the dataset used included 4998 images of benign (CB) colon tissues and 5000 images of colon adenocarcinomas (CA). Table 2 shows a class-wise distribution of benign colonic tissue and colon adenocarcinoma images in the training and testing sets for each fold of the five-fold stratified cross-validation.

### 4.2. Hardware, Software and Experimental Settings

All research investigations were performed on a PC with the Apple Mac Studio (2023) architecture, which includes an Apple M2 Ultra CPU, 64 GB of RAM, a 1 TB SSD, and a 32-core GPU. The computer operated using macOS Sequoia version 15.6.1 (Apple Inc., Cupertino, CA, USA).

The programming environment utilized was Python (3.10.19), along with the following primary libraries: Pandas (2.3.3), Scikit-learn (1.7.2), SHAP (0.49.1), Matplotlib (3.10.8), TensorFlow (2.19.1), OpenCv (4.12.0.88), ImageHash (4.1.0), TQDM (4.67.x), and Pillow (8.4.0).

The augmentation methods consist of random rotation (±25°), color jitter (brightness of ±30%, contrast of ±30%, saturation of 30%, hue of 0.05).

The experiments were developed in the following conditions: The hyperparameters for the training procedure were defined as a batch size of 16, a learning rate of 0.001, 20 epochs, and the input size of images at 224 × 224 × 3, Adam optimizer with TensorFlow/Keras default parameters, binary cross-entropy loss function, Dense(256, ReLU) → Dropout(0.3) → Dense(64, ReLU) → Dense(1, Sigmoid) classification-head architecture, all pre-trained CNN backbone layers were frozen, no model checkpointing, early stopping, or best-epoch selection criterion was implemented, and data augmentation was applied only to the training folds.

The 70/30 train–test split for each fold was performed before any data augmentation. The results were assessed by 5-fold cross-validation to guarantee the accuracy and stability of the classifications. To ensure reproducibility, all random operations, including the train/test split and 5-fold stratified cross-validation, were performed using a fixed random seed of 42.

### 4.3. Pretrained CNNs

The pre-trained Convolutional Neural Networks (CNNs) are deep learning (DL) models that have typically been trained on ImageNet and saved along with their learned weights. These DL models are efficient because they reduce runtime and data size, support the knowledge transfer, and are adaptive using the transfer learning process [29].

**Figure 3:**
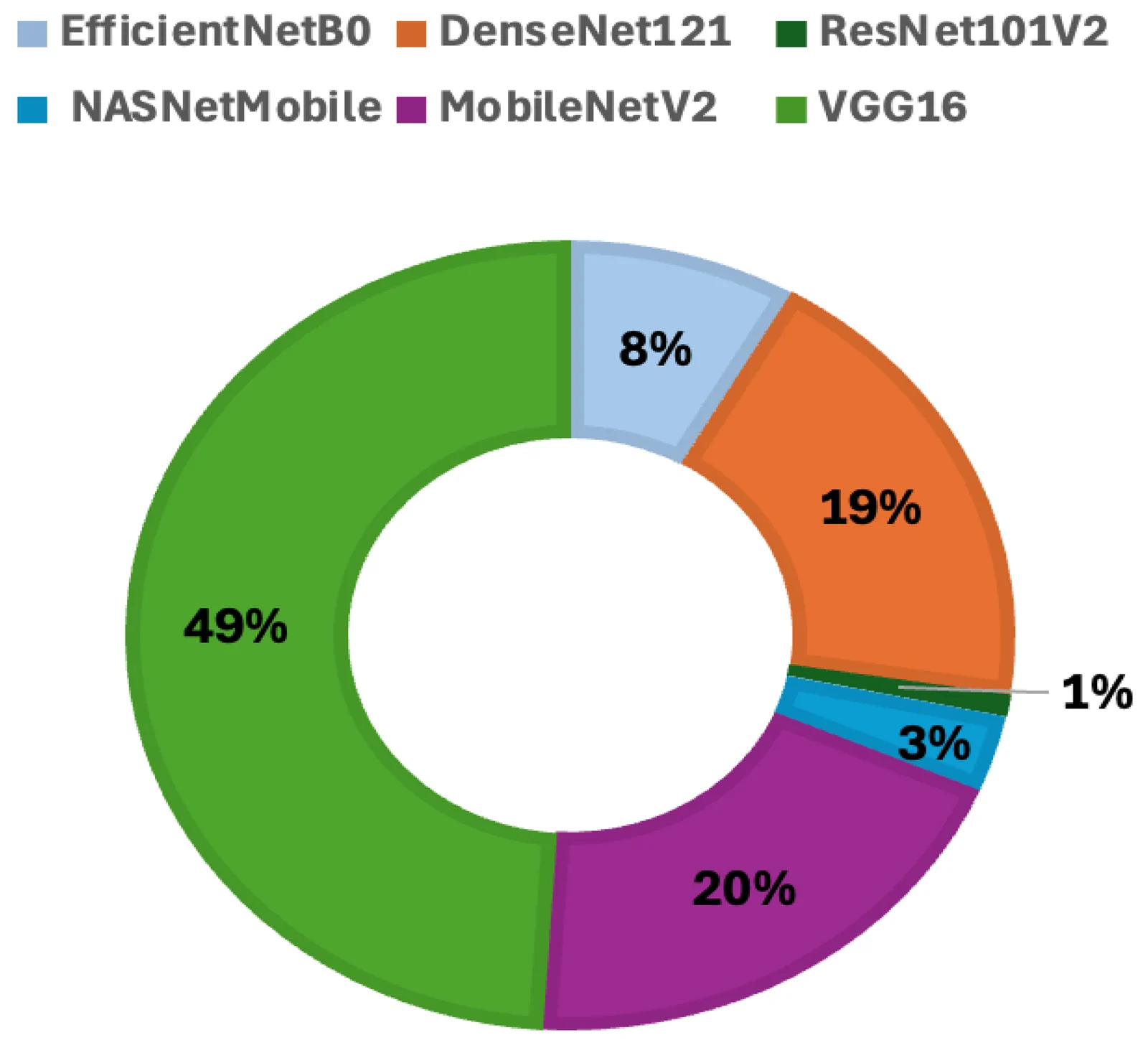
The percentage of published papers that have used pre-trained convolutional neural networks (CNNs) over the past five years (2021–2025).

When utilizing a pre-trained CNN in a study, it is essential to verify its applicability based on the type of images being analyzed. To this end, the selection of pre-trained CNNs—including EfficientNetB0, DenseNet121, ResNet101V2, NASNetMobile, MobileNetV2, and VGG16—was conducted through a search on the Google Scholar platform, using the keywords “histopathologic images” and “pre-trained CNN.” The six best CNNs were found, and the number of times the phrases “histopathologic images” and “name CNN” appeared was counted. “Name CNN” was replaced with each of the previously chosen models. Figure 3 presents a statistical overview of the usage of these pre-trained models over the past five years, obtained on 15 January 2026.

For each deep learning model, the numbers of parameters and layers, input image size, and model size are stored in Table 3.

### 4.4. Machine Learning Algorithms

Random Forest and Histogram-based Gradient Boosting machine learning classifiers were selected for classifying handcrafted features through five cross-validation processes. They were chosen because they are compatible with SHAP tools.

Random Forest constructs multiple decision trees and aggregates their predictions to classify data through a majority vote. In this model, the number of decision trees in the forest is set to 500, which helps mitigate overfitting that can occur when a single decision tree is used.

Histogram-based HGB’s gradient boosting is an improved version of traditional gradient boosting that is faster and uses less memory. It also uses the same sequential boosting idea as regular gradient boosting, but it groups features into separate histograms, which cuts down on the number of possible split points. In this study, the maximum depth of each tree is set to 6, the learning rate that governs the contribution of each tree is 0.5, and there is a total of 300 boosting iterations.

### 4.5. Explainable Artificial Intelligence

To explain Random Forest and Histogram-based Gradient Boosting’s predictions for the positive class, the SHAP method was proposed. This shows the overall importance of features and their impact on prediction. An observation from the summary plot of SHAP means on the x-axis is the impact on the model output and on the y-axis the features sorted by importance [36].

Gradient-weighted Class Activation Mapping (Grad-CAM), used with preponderance in medical imaging, provides visual explanations for pre-trained CNN predictions by highlighting important regions in the input image that influence the model’s decision. This acts on the last convolutional layer where, because it retains spatial information and has high-level features, it computes gradients of the target classes, colon adenocarcinoma and benign, respecting the feature maps [37].

Explainable AI is important in medical applications. The effectiveness of deep learning (DL) models relies on data quality, dataset size, and processing resources. While significant handcrafted features extracted from medical imaging can enhance the power of a DL model, older methods such as radiomics features may sometimes be more appropriate and often offer better explainability [38]. A comprehensive and recent review, a landmark for XAI in cancer care, was proposed by Khurana et al. [39]. It evaluates current methods, challenges, regulatory considerations, and future strategies for creating transparent and clinically trustworthy AI systems in oncology.

### 4.6. Handcrafted Features

In histopathology, diagnosis relies on the analysis of tissue architecture, gland formation, and cellular irregularity. The presence of CC alters these patterns, which can be quantified using features such as Shannon entropy, Gini index, and radius of gyration. The second dataset utilized in hybrid decision-level fusion comprises handcrafted features derived from insights gained through the texture of histopathologic images.

These features explicitly extract measurable properties of an image, such as edges, textures, shapes, patterns, and colors. In this study, we focused on characterizing patterns because histopathological images are composed of repetitive clusters of cells [40].

#### 4.6.1. Shannon Entropy

Shannon entropy (SE) is commonly used in image processing as a way to measure texture complexity. This measure relies on the probability distribution of pixel intensity values, which is usually obtained from an image histogram [41,42].

(1) $$\mathrm{SE}=-\sum_{i=0}^{N-1}p_{i}logp_{i}$$

#### 4.6.2. Gini Index

The Gini Index (GI) is a statistical measure used to assess inequality, dispersion, pattern density, or homogeneous tissue versus heterogeneous tissue. In pattern recognition and image analysis, this method is modified to assess the uneven distribution of pixel intensities, textures, or features within an image or a specific area.

(2) $$GI=\frac{2\sum_{i=1}^{n}ix_{i}}{n\sum_{i=1}^{n}x_{i}}-\frac{n+1}{n}$$

where $x_{i}$ pixel intensity, I rank index, *n* number of pixels, and $\left(n+1\right)/n$ normalization term [43].

#### 4.6.3. Radius of Gyration

The radius of gyration (RG) serves as a descriptor for shape and spatial distribution. In the context of image processing, it is used to explain cell morphology or particle clustering; it quantifies the extent to which pixels or features are distributed from a reference point, typically the centroid of an object or region [44].

(3) $$\mathrm{RG}=\sqrt{\frac{1}{N}\sum_{i=1}^{n}{\left(x_{i}-x_{c}\right)}^{2}+{\left(y_{i}-y_{c}\right)}^{2}}$$

where $\left(x_{i},y_{i}\right)$ are coordinates of pixel and $\left(x_{c},y_{c}\right)$ centroid of the region.

### 4.7. Late Fusion

Late fusion is utilized in hybrid decision-level fusion medical AI to merge deep features with structured or handcrafted features. This approach offers a notable advantage in that the CNN and ML models independently generate predictions before combining them.

The approach provides flexibility because models can be combined without needing to retrain them jointly, resulting in high performance. Additionally, it is straightforward to add or remove models from the fusion pipeline. Equation (4) combines the probabilities, P_CNN_ and P_ML_, provided by the CNN and ML models, respectively [45]. The variable α represents the fusion weight, which is tuned using values of 0.4, 0.5, and 0.6.

(4) $$P_{final}=\alpha P_{CNN}+(1-\alpha )P_{ML}$$

The α indicates the level of importance assigned to each modality (P_CNN_, P_ML_) during the combination of weights. The fusion weight was only selected on the validation folds in the training set during the five-fold cross-validation procedure. Parameter selection was not based on the independent test set.

## 5. Results and Discussions

This paper aims to classify CC more accurately using late fusion techniques involving EfficientNetB0, DenseNet121, ResNet101V2, NASNetMobile, MobileNetV2, and VGG16, as well as RF and HGB ML classifiers. This study utilized the LC25000 colon histopathological image dataset along with handcrafted pattern features. The late fusion process comprised four main components, the first of which involved the selection of the histopathological image dataset.

The second step involves a rigorous selection of pretrained CNNs and MLs, with the rationale for this choice outlined in Section 4.3 and Section 4.4.

To validate the enhancement provided by handcrafted features in hybrid decision-level fusion, we computed the performance of standalone CNNs; the results are presented in Table 3. To demonstrate the effectiveness of the prediction model for CC, we evaluated all selected CNNs in conjunction with RF and HGB, respectively, using five-fold cross-validation. The final two steps focus on obtaining and explaining the results through a final fusion (refer to Equation (4)), following a fine-tuning of α. The quality metrics are shown in Table 4 when the tuning was established at α = 0.5, in accordance with Equation (4); the deep features extracted from the image account for 50% of the contribution, while handcrafted features account for 50%. The table presents the accuracy, F1-score, MCC, and AUC metrics for all combinations of CNNs and MLs.

When α = 0.6, the recorded values of quality metrics showed a decrease: accuracy decreased by 1.2%, the F1-score decreased by 1.04%, the MCC decreased by 1.06%, and the AUC decreased by 0.96%.

For α = 0.4, the recorded values of quality metrics also showed decreases: accuracy decreased by 0.97%, the F1-score decreased by 0.89%, the MCC decreased by 0.78%, and the AUC decreased by 0.56%.

The SHAP and Grad-CAM XAI methods are essential because CNN and ML models need to be trusted and validated. It is important to understand the reasoning behind their decisions after training. We introduced SHAP to identify which features influence predictions and to clarify the contribution of each handcrafted feature.

**Figure 4:**
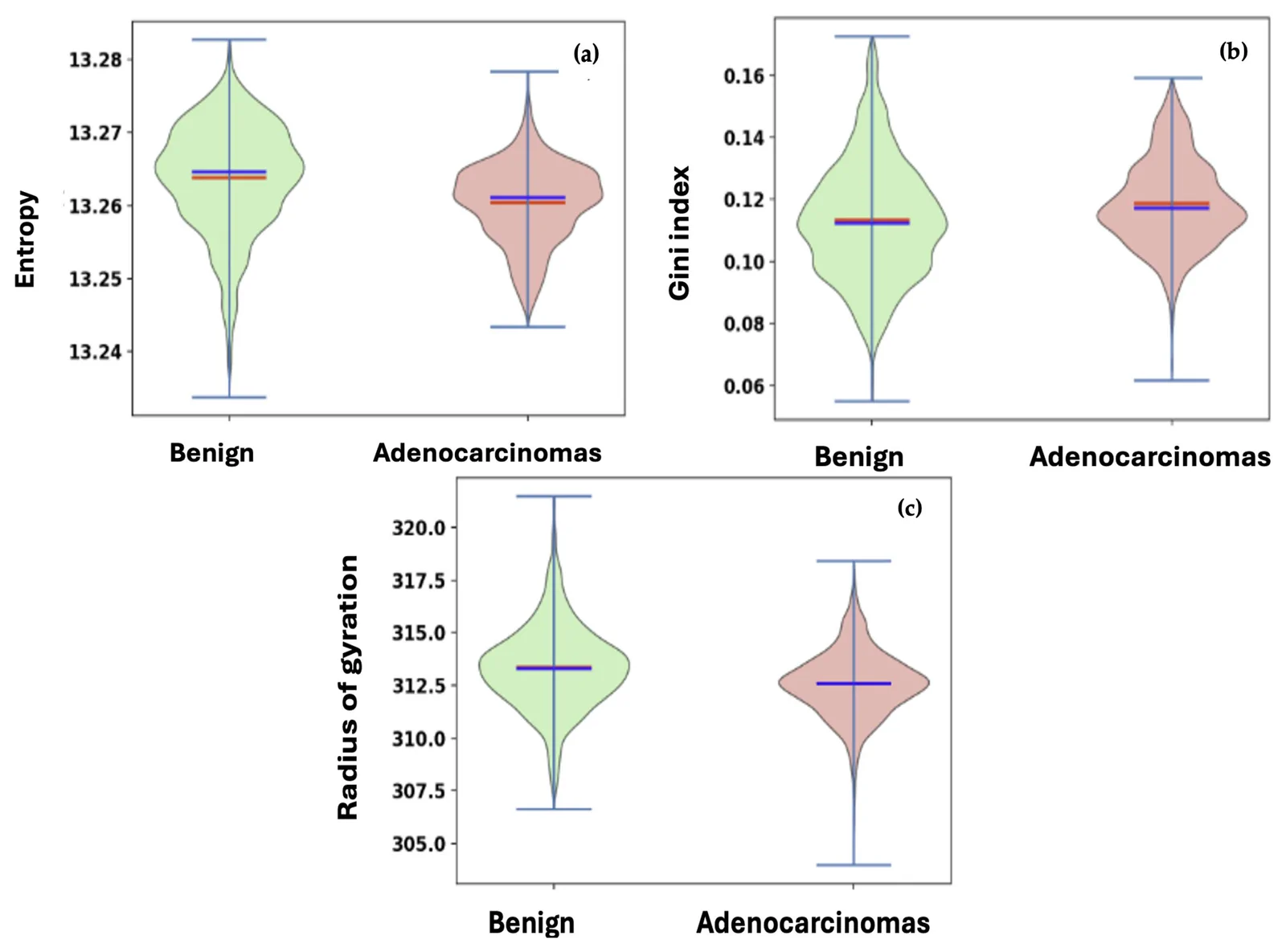
Violin plots of handcrafted features: (**a**) entropy; (**b**) Gini index; (**c**) radius of gyration.

The handcrafted features used as the second set in hybrid decision-level fusion are distinctive; consequently, we represent them in Figure 4 using violin plots. Entropy, the Gini index, and the radius of gyration stand out as key handcrafted features in our analysis that compare benign and adenocarcinomas. In the violin plot, the minimum and maximum values are indicated, with a red line representing the median (the middle data point) and a blue line indicating the mean (the average value). When comparing the two classes, it is evident that the benign data exhibit greater variability than the adenocarcinomas across all handcrafted features.

Table 4 and Table 5 present the key results from this study, focusing on accuracy, F1-score, MCC, and AUC. The experimental outcomes are reported as mean ± standard deviations, calculated following a five-fold cross-validation process. Each fold of the training process utilized the proposed pretrained CNNs with the number of images specified in Table 2. It is important to note that Table 4 displays the quality metrics for standalone CNNs, while Table 5 presents the results for each hybrid decision-level fusion model.

The comparison between the performance of all standalone CNNs and all hybrid decision-level fusion models of RF with HGB showed an increase in average accuracy of 1.6%, an F1-score increase of 1.8%, an MCC increase of 2.7%, and an AUC increase of 1.1%.

For DenseNet121, the integration with RF increased the accuracy from 0.921 to 0.935, the F1-score from 0.920 to 0.934, the MCC from 0.842 to 0.870, and the AUC from 0.968 to 0.978. Similarly, ResNet101V2 combined with RF achieved the best overall performance, improving the standalone accuracy from 0.928 to 0.941 and increasing the MCC from 0.856 to 0.883. These results reflect the boost that handcrafted features bring to hybrid decision-level fusion.

To evaluate the contribution of each handcrafted feature category, an ablation study was conducted by removing one feature at a time while keeping the classifier and training protocol unchanged. Systematically handcrafted features from the initial set: entropy, Gini index and radius of gyration were removed. The graphs (a) to (f) in Appendix A show the performance of combination (a) entropy; (b) Gini index; (c) radius of gyration; (d) entropy and radius of gyration; (e) radius of gyration and Gini index; and (f) entropy and Gini index.

**Figure 5:**
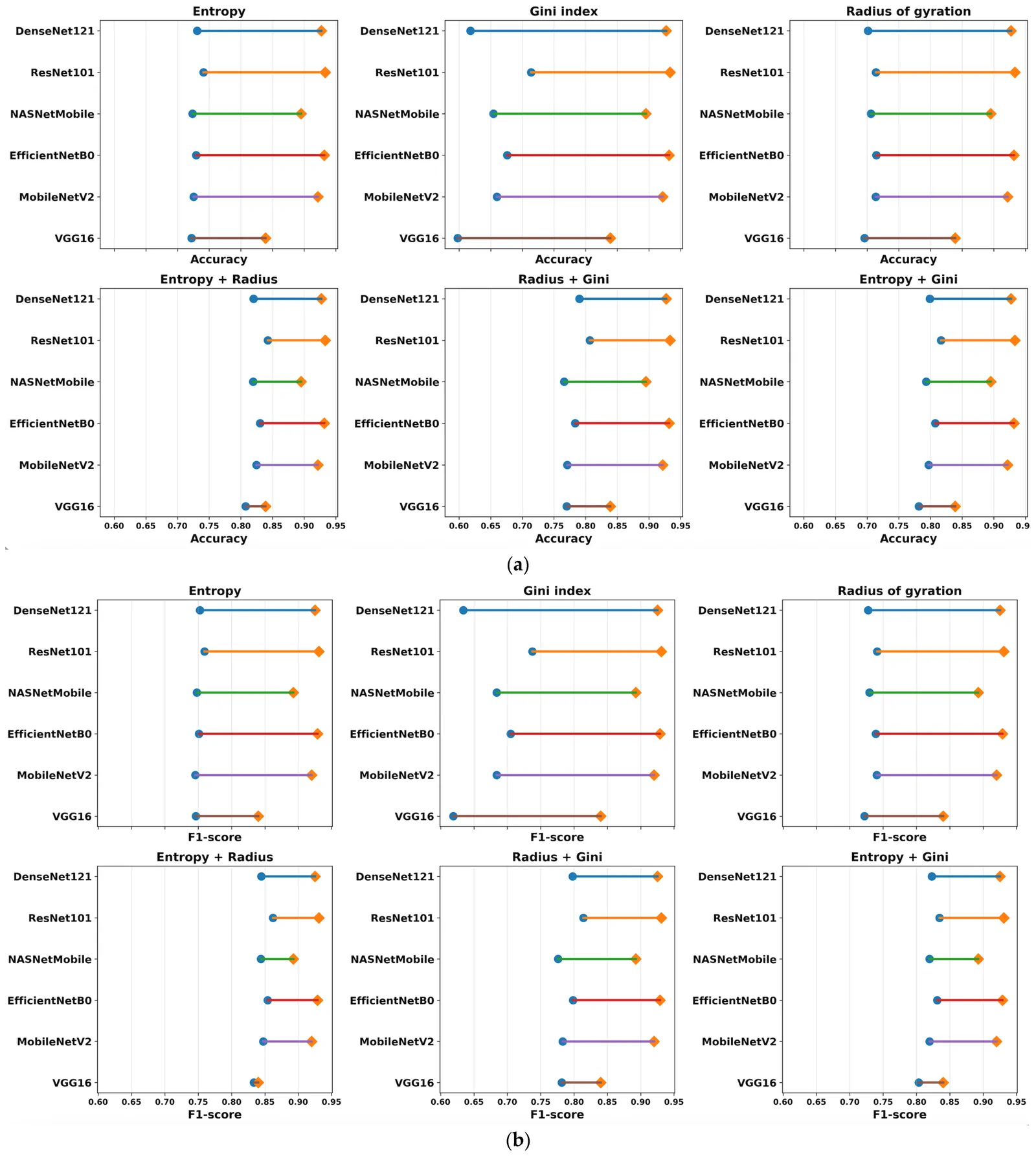

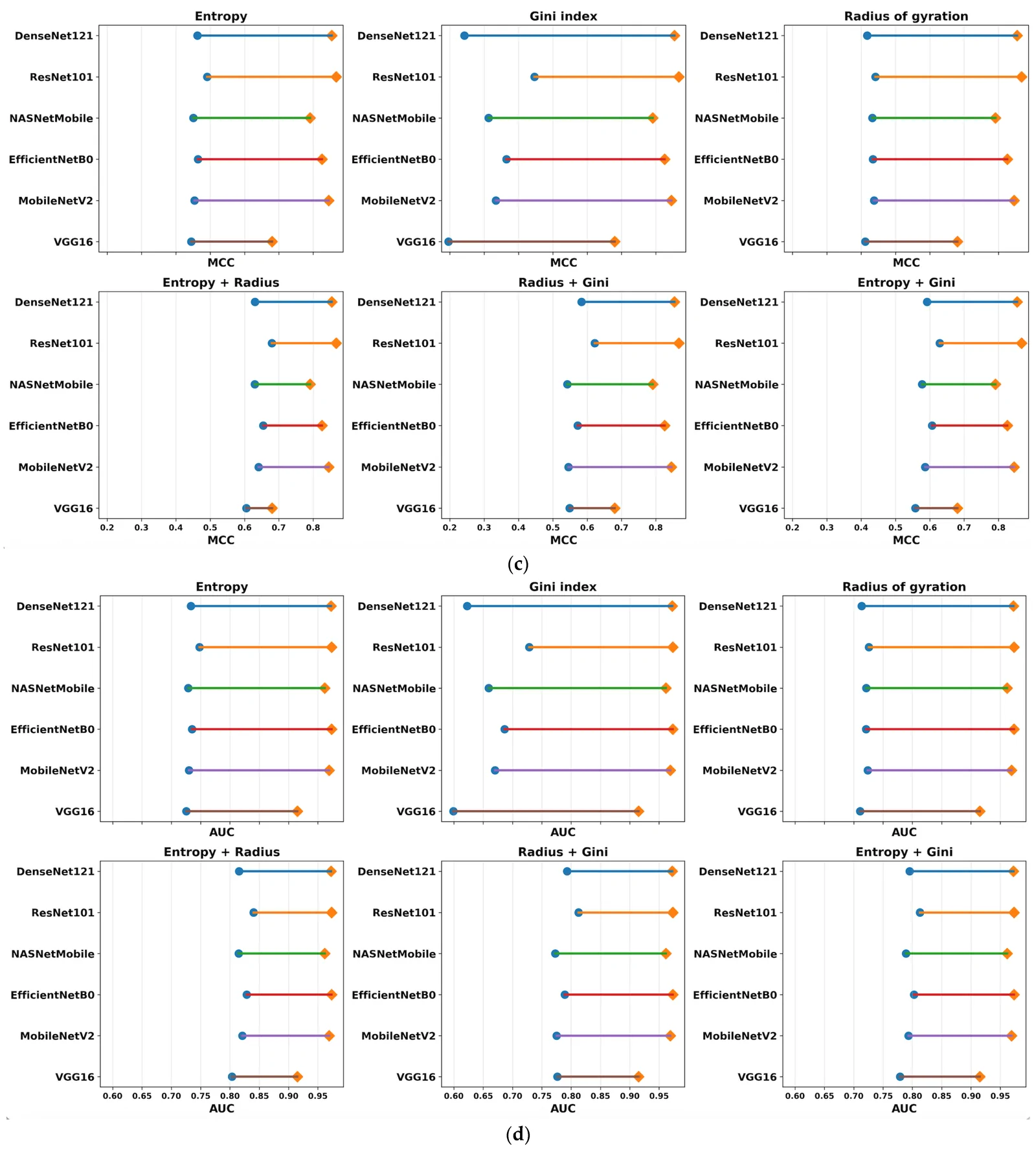
Performance degradation after feature ablation across different CNN backbones: (**a**) Accuracy; (**b**) F1-score; (**c**) MCC; (**d**) AUC.

The performance differences between the full feature set (Table 5) and the six ablation configurations (Appendix A) are illustrated in Figure 5 by means of dumbbell plots. This compares the complete results set (Table 5) with each ablation configuration (Appendix A) using the average performance of the RF and HGB classifiers. Diamond markers denote the full model, and circular markers denote the ablation models. The length of every connecting segment indicates the extent of performance degradation when removing one or more handcrafted features.

In this way, we observe their impact on model performance: removing handcrafted features causes a decline in performance, indicating that they are highly informative when used together.

To quantitatively assess the stability of each CNN in the ablation process, the Robustness Index (RI) was computed as $RI=1-\bar{\Delta }$, where $\bar{\Delta }$ is the average dumbbell-segment length across all six ablation configurations. Higher RI values indicate smaller performance degradation after feature removal and therefore greater robustness. In the following, the model and the significance of the RI are mentioned. VGG16 achieved the highest robustness (RI = 0.849), indicating that its performance was least affected by feature ablation; DenseNet121 exhibited the lowest robustness (RI = 0.765), suggesting a stronger dependence on the complete feature set. NASNetMobile (RI = 0.802) and ResNet101V2 (RI = 0.800) also demonstrated high robustness; both EfficientNetB0 (RI = 0.788) and MobileNetV2 (RI = 0.778) showed intermediate sensitivity to feature removal.

The complete model always gives the best values of accuracy, F1-score, MCC, and AUC for all the CNN backbones. As expected, the removal of handcrafted descriptors leads to a decrease in classification performance, with the magnitude of the decrease depending on the removed feature. The least degradation is generally observed after removal of the entropy descriptor and removal of the Gini index or combinations of two descriptors result in much larger decreases. These findings suggest that the handcrafted descriptors provide complementary information to the deep features extracted by the CNN backbones and that their joint use significantly boosts the consistent performance and discriminative power of the proposed classification framework.

The hybrid decision-level fusion process involved a step-by-step empirical approach where deep features were fused with handcrafted features. The sets of handcrafted features included entropy, Gini index, radius of gyration, entropy and Gini index, entropy and radius of gyration, Gini index and radius of gyration, and the combination of entropy, Gini index, and radius of gyration. Table 5 highlights the best results achieved with the last combination. Appendix A stores all results regarding the mentioned experiments.

**Figure 6:**
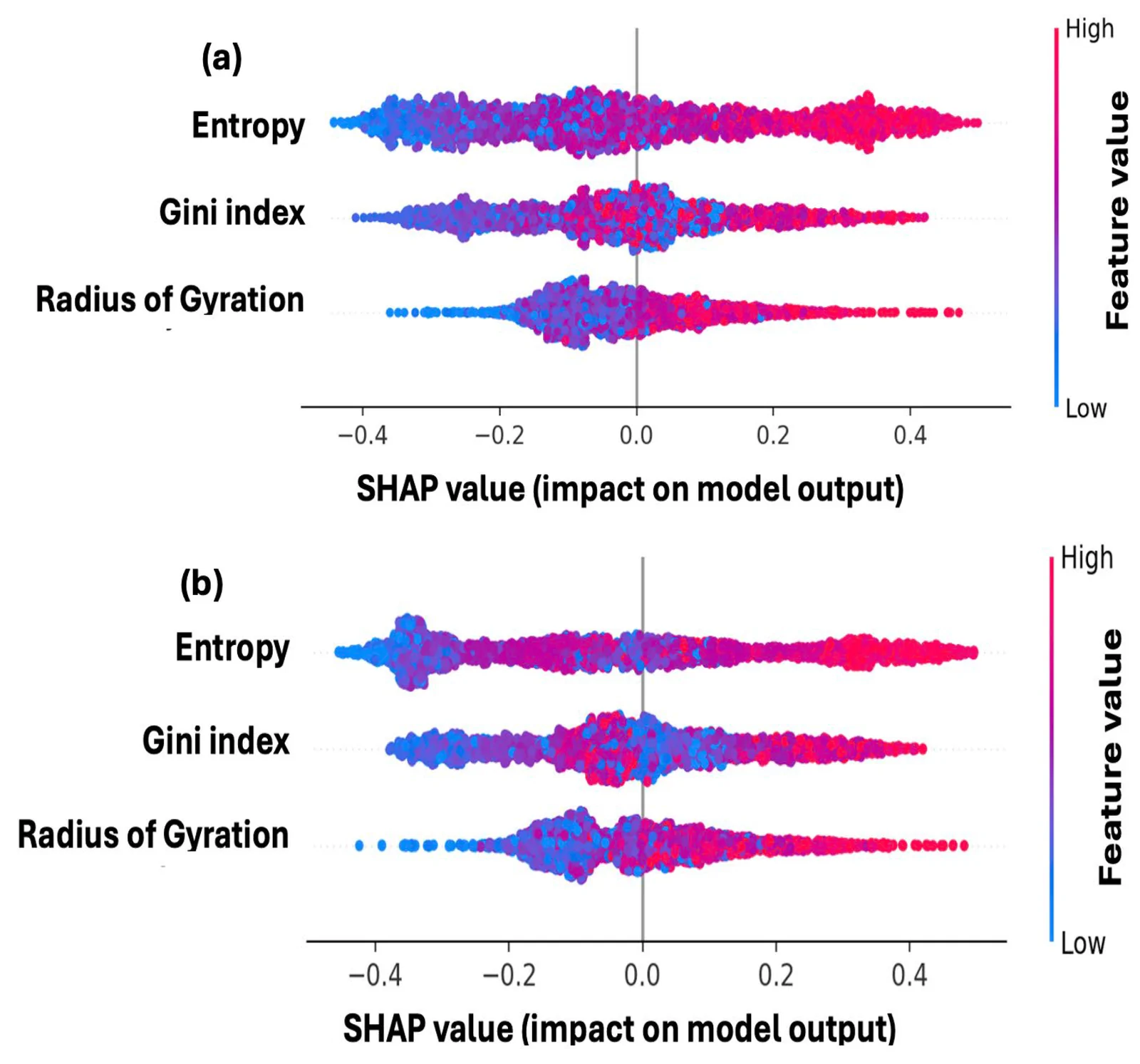
Comparison of handcrafted feature contributions (**a**) Random Forest; (**b**) Histogram-based Gradient Boosting.

The SHAP method efficiently extracts feature contributions and computes the average model output over the training data. In Figure 6, a distinct interpretation for handcrafted features is presented for RF and HGB. It provides insights into how the contribution of an individual feature to the model output is influenced by its value. The position of each dot indicates the feature’s impact on the model, while the color of the dot reflects the value of that feature in the review. Figure 6 shows a notable difference between features, with entropy identified as the most crucial feature for both RF and HGB. The impact on prediction is also attributed to entropy, as indicated by the density of red points associated with this feature. In comparison to the representation in Figure 4, the raw data for entropy is denser above the median line compared to the Gini index and radius of gyration.

**Figure 7:**
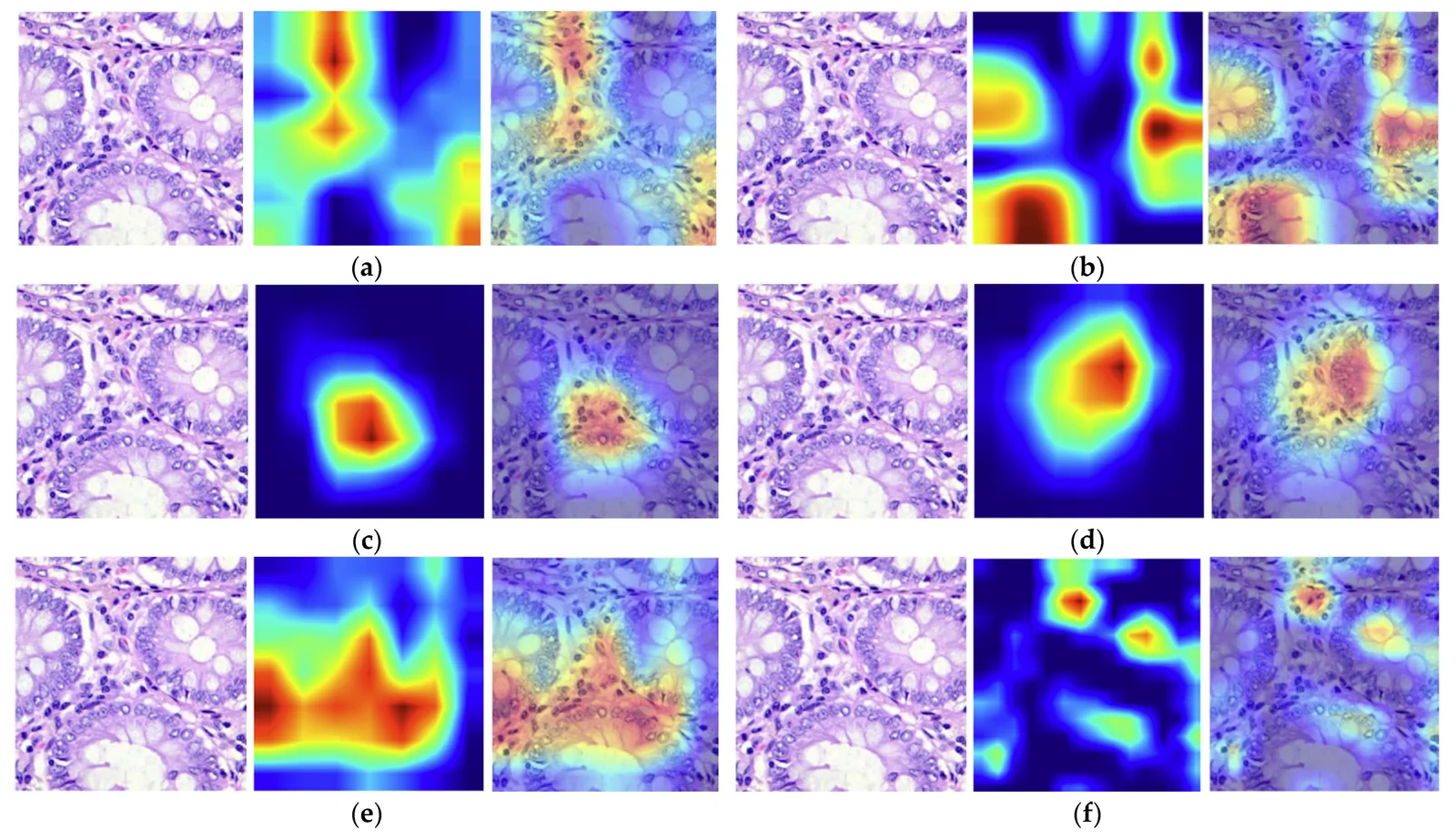
Grad-CAM heatmap analysis: (**a**) EfficientNetB0; (**b**) DenseNet12; (**c**) ResNet101V2; (**d**) NASNetMobile; (**e**) MobileNetV2; (**f**) VGG16.

Grad-CAM is a common technique for visualizing feature maps, employing gradients to assess the relevance of spatial locations within the final convolutional layer of each CNN. In the heat map produced by Grad-CAM, blue indicates the inconsequential area, whereas red denotes the important region linked to that category. The model makes decisions based on the previously mentioned local pixel-level details highlighted in red and yellow. For the visual comparison from Figure 7 to be valid, it shows the Grad-CAM results obtained for the same histopathological image generated for the DenseNet121, ResNet101V2, NASNetMobile, EfficientNetB0, MobileNetV2, and VGG16 models.

The Grad-CAM visualizations highlight noteworthy differences between the proposed CNN in terms of the capacity to highlight clinically relevant regions. All models mainly target epithelial tissue, although there are significant differences in the accuracy, spatial coverage, and consistency of the activation maps. In the following, the identified regions are discussed for each model. ResNet101V2 provides the most discriminative visualization, focusing its attention on a small epithelial area containing densely packed hyperchromatic nuclei and an irregular glandular architecture, both well-known histopathological hallmarks of colorectal adenocarcinoma. EfficientNetB0 also shows good localization, highlighting the epithelial bridges between neighboring glands while efficiently excluding the glandular lumina, thus capturing cellular morphology and glandular organization. NASNetMobile highlighted a comparable problematic region, but with a larger activation area that includes more tissue background, which may help identify glandular linkages. The DenseNet121 yields more diffuse activations over many contiguous glands. MobileNetV2 activates over border areas of the epithelium, indicating a greater dependence on global texture features rather than localized cellular defects. VGG16 is the least informative, producing fragmented hotspots scattered across the tissue without clearly outlining a contiguous diseased region.

In our study, we integrated handcrafted features that characterize the spatial arrangement of pixels and extracted information from pathology. This approach introduces a novel method for identifying the most effective models applicable to histopathological images. This late fusion allows our model to leverage both image-level patterns and embedded information guidance.

The obtained results indicate that the late fusion approach enhances performance compared to other baselines. This improvement is evident through comparisons with state-of-the-art studies listed in Table 6. Comparisons with the results obtained on the LC25000 dataset were performed.

The comparisons were made with the state-of-the-art papers that used only CNN models [45,47,49,50] and with the papers that integrated hybrid decision-level fusion [47,48]. Our results are comparable to these, surpassing the results obtained in [47,50] and are approximately equal to the findings from studies [45,50]. Additionally, the studies employing multimodal learning [46,48] slightly surpassed the results obtained in this study. This means that, besides providing the best results, the hybrid decision-level fusion system more effectively integrates complementary sources of information, such as visual and textual data, which allows for a more comprehensive understanding and processing of complex tasks.

Three major limitations were detected during the study: (i) all experiments were conducted on a single public dataset, namely the LC25000; (ii) the transfer and multimodal learning, where two datasets are analyzed with six CNNs and two MLs, is time-consuming; and (iii) the limited number of handcrafted features.

## 6. Conclusions

To summarize, the combination of deep learning techniques and handcrafted features is a significant step forward in achieving accurate classification of benign and adenocarcinoma colon cancer. This study highlights the effectiveness of the hybrid decision level when combining ResNet101V2 and RF, achieving an accuracy of 94.1%. This approach in the hybrid decision-level process provides a trusting look into the future of the classification of different digital pathologies; moreover, integrated AI systems can assist pathologists in delivering more accurate, data-driven diagnoses with greater efficiency and confidence. The proposed complete pipeline includes data extraction, pre-analysis using violin graphs, application of transfer learning, classification of handcrafted features, and validation of results through the XAI method, which enhances the trustworthiness of the study.

Future studies will concentrate on the classification of various histopathologic images associated with different pathologies. Furthermore, these studies will incorporate a significant number of handcrafted features into the deep feature system. This approach aims to further refine diagnostic accuracy and improve the overall efficiency of the diagnostic process.

## Figures and Tables

**Table 1:** Comparisons with cutting-edge papers.

References	Model 1	Model 2	XAI	Dataset	Quality Metric
Shirae et al. (2025) [15]	DenseNet201 VGG11_bn VGG19_bn ResNet50d ConvNeXtV2	Transformers: GCViT, CoAtNet BEiTV2	no	TCGA Gliomas	Acc. 93.6% AUC 96.7%
Jemane et al. (2025) [16]	VGG16	Random Forest	no	Ethiopian Jimma Medical Center	Acc. 91%
Ma et al. (2025) [17]	ResNet18	Pathology Mutual Guidance Fusion Diagnostic Network	no	WSIs	Acc. 83.63% AUC 75.45%
Zhou et al. (2025) [18]	ResViT-GANNet	TransMIL, CtransPath, SwinCNN	yes	BreakHis, BACH	Acc. 96.40% Precision 96.34% Recall 96.36% F1-score 96.35%
Byeon et al. (2025) [19]	Convolutional Neural Networks (CNNs)	Multi-View Transformer Online Fusion Mutual Learning	yes	BreakHis	Acc. 90%
Ferber et al. (2024) [20]	Generative Pretrained Transformer 4	kNN	no	CRC100K	Acc. 61.7%
Wang et al. (2024) [21]	SHAP-CAT	Random Forest, Logistic Regression, SVM, Decision Tree (DT), KNN	yes	IHC4BC-PR	Acc. 99.7% AUC 95%
Dwivedi et al. (2022) [22]	CNN	Graph neural networks	no	WSIs	Cohen’s kappa 71%
Mewada et al. (2024) [23]	DenseNet161 spatial features	DenseNet161 residual features	no	BreakHis	Acc. 94.65%
Ahmad et al. (2023) [24]	Inceptionv3, InceptionResNetV2, NASNetLarge, and DenseNet201	SVM	no	OSCC	Acc. 98.92%,
Yang et al. (2022) [25]	ResNet50	RF	no	The Cancer Genome Atlas (TCGA)	AUC 76%
Rohini et al. (2023) [26]	VGG19 DenseNet201	Customized CNN	no	Brain MRI Images for Brain Tumor Identification	Acc. 99.43% sensitivity 98.73%, specificity 97.21%
Tătaru et al. (2025) [4]	Custom-Built CNN	PyCaret Auto Machine Learning	no	University of Medicine and Pharmacy, Iași, Romania	Acc. 94.5%
Attallah (2025) [27]	EfficientNetB0, MobileNet, and ResNet-18	DT, SVM, KNN	no	Lung and Colon Cancer LC25000.	specificity 99.7% precision 99.78%

**Table 2:** Sample distribution for five-fold stratified cross-validation.

	Training			Testing		
Fold	CB	CA	Total	CB	CA	Total
Fold 1	2799	2800	5599	700	700	1400
Fold 2	2799	2800	5599	700	700	1400
Fold 3	2799	2800	5599	700	700	1400
Fold 4	2799	2800	5599	700	700	1400
Fold 5	2800	2800	5600	699	700	1399

**Table 3:** Key specifications for used pre-trained CNNs.

Deep Learning Model	Number of Parameters (Millions)	Number of Layers	Input Image Size (RGB)	Model Size (MB)
EfficientNetB0 [30]	~5.3	237	224 × 224 × 3	~20
DenseNet121 [31]	~8	121	224 × 224 × 3	~32
ResNet101V2 [32]	~44.5	101	224 × 224 × 3	~170
NASNetMobile [33]	~5.3	88	224 × 224 × 3	~23
MobileNetV2 [34]	~3.4	88	224 × 224 × 3	~14
VGG16 [35]	~138	16	224 × 224 × 3	~528

**Table 4:** Quality metrics for stand-alone CNNs.

Deep Learning Model	Accuracy	F1-Score	MCC	AUC
DenseNet121	0.921 ± 0.012	0.920 ± 0.008	0.842 ± 0.016	0.968 ± 0.005
ResNet101V2	0.928 ± 0.009	0.927 ± 0.007	0.856 ± 0.014	0.971 ± 0.004
NASNetMobile	0.887 ± 0.014	0.885 ± 0.015	0.775 ± 0.026	0.955 ± 0.006
EfficientNetB0	0.926 ± 0.008	0.924 ± 0.007	0.848 ± 0.013	0.972 ± 0.004
MobileNetV2	0.914 ± 0.011	0.913 ± 0.012	0.828 ± 0.020	0.962 ± 0.005
VGG16	0.826 ± 0.016	0.824 ± 0.017	0.651 ± 0.038	0.901 ± 0.007

**Table 5:** Quality metrics for each hybrid decision-level fusion model.

Deep Learning Models	ML	Accuracy	F1-Score	MCC	AUC
DenseNet121	RF	0.935 ± 0.008	0.934 ± 0.008	0.870 ± 0.018	0.978 ± 0.004
	HGB	0.919 ± 0.010	0.916 ± 0.010	0.838 ± 0.023	0.967 ± 0.005
ResNet101V2	RF	0.941 ± 0.007	0.940 ± 0.007	0.883 ± 0.019	0.979 ± 0.004
	HGB	0.925 ± 0.009	0.922 ± 0.009	0.851 ± 0.020	0.968 ± 0.005
NASNetMobile	RF	0.903 ± 0.011	0.902 ± 0.011	0.807 ± 0.029	0.967 ± 0.005
	HGB	0.887 ± 0.014	0.883 ± 0.013	0.775 ± 0.032	0.956 ± 0.006
EfficientNetB0	RF	0.940 ± 0.007	0.938 ± 0.007	0.827 ± 0.045	0.979 ± 0.004
	HGB	0.923 ± 0.009	0.920 ± 0.010	0.824 ± 0.045	0.968 ± 0.006
MobileNetV2	RF	0.930 ± 0.008	0.929 ± 0.008	0.861 ± 0.018	0.975 ± 0.004
	HGB	0.913 ± 0.011	0.911 ± 0.010	0.829 ± 0.020	0.963 ± 0.005
VGG16	RF	0.847 ± 0.021	0.849 ± 0.020	0.696 ± 0.025	0.921 ± 0.006
	HGB	0.831 ± 0.023	0.831 ± 0.023	0.664 ± 0.024	0.909 ± 0.007

**Table 6:** Comparisons with cutting-edge papers.

References/Year	Deep Learning Model	Dataset	Training/Testing	Metric Quality
Degadwala and Oza (2025) [46]	VGG16	LC25000	80/20	94% accuracy
Ochoa-Ornelas et al. (2024) [47]	MobileNetV2 EfficientNetB3 and Gray Wolf Optimizer (GWO)	LC25000 + National Cancer Institute GDC Data Portal	80/20	94.8% F1-score
Gül (2025) [48]	ResNet50	LC25000 + Local Binary Pattern	70/30	67.53% accuracy
Hasan et al. (2025) [49]	iBOT and MLP	LC25000	70/30	96.1% accuracy
Karegoudra et al. (2025) [50]	DenseNet	LC25000	70/30	94.3% accuracy
Bukhari et al. (2020) [51]	ResNet-18	LC25000	60/40	93.04% accuracy
Proposed method	ResNet101V2 and RF	LC25000+ handcrafted features	70/30	94.1% accuracy 94% F1-score

**Figure A1:**
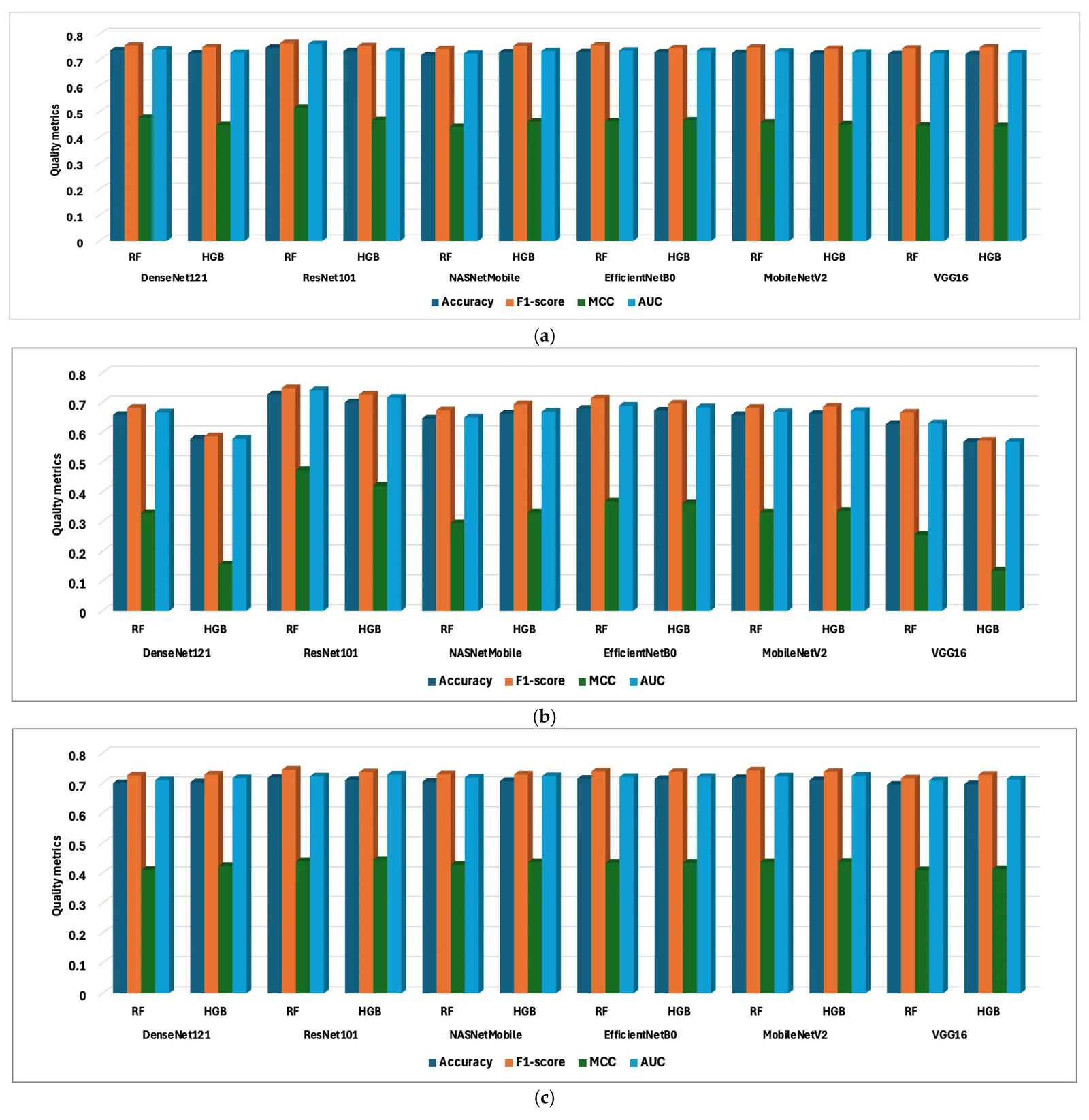

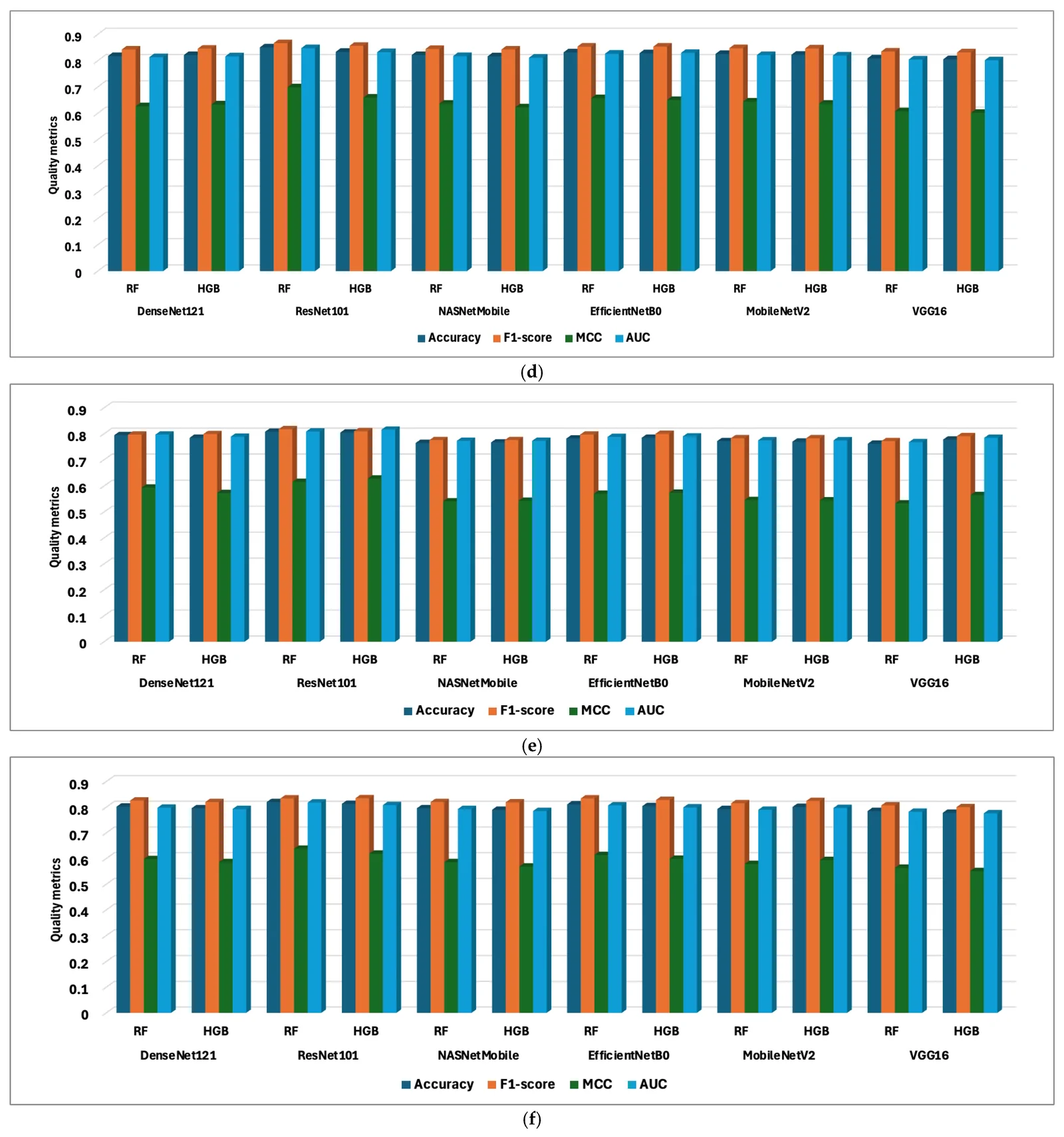
Evaluation of hybrid decision-level fusion based on deep features and handcrafted features: (**a**) entropy; (**b**) Gini index; (**c**) radius of gyration; (**d**) entropy and radius of gyration; (**e**) radius of gyration and Gini index; (**f**) entropy and Gini index.

## Data Availability

The data that support the findings of this study are openly available at https://github.com/tampapath/lung_colon_image_set (accessed on 22 December 2025), and the tabular data at https://github.com/simonamoldovanu/pattern_features_lung (accessed on 10 March 2026).

## Abbreviations

The following abbreviations are used in this manuscript:

CNN	Convolutional Neural Networks
HGB	Histogram Gradient Boosting
ML	Machine Learning
RF	Random Forest
CC	Colon Cancer
AUC	Area Under the Curve
Grad-CAM	Gradient-weighted Class Activation Mapping
SHAP	SHapley Additive exPlanations
MCC	Matthews Correlation Coefficient
SVM	Support Vector Machine
KNN	k-Nearest Neighbors
XAI	Explainable AI
SE	Shannon Entropy
GI	Gini Index
RG	Radius of Gyration
CA	Colon Adenocarcinoma
CB	Colon Benign
